# The complete chloroplast genome of *Bambusa ventricosa* (Bambusoideae: Bambuseae)

**DOI:** 10.1080/23802359.2018.1507645

**Published:** 2018-08-23

**Authors:** Xianzhi Zhang, Renchao Zhou, Siyun Chen

**Affiliations:** a School of Life Sciences, Sun Yat-sen University, Guangzhou, Guangdong, China;; b Germplasm Bank of Wild Species, Kunming Institute of Botany, Chinese Academy of Sciences, Kunming, Yunnan, China

**Keywords:** Chloroplast genome, *Bambusa ventricosa*, phylogenomics

## Abstract

*Bambusa ventricosa* is one important ornamental bamboo. In this study, we generated the complete chloroplast (cp) genome of *B. ventricosa* via genome-skimming method. The complete cp genome is 139,460 base-pairs (bp) in size, with a pair of inverted repeats of 21,794 bp which separate a large single copy region of 82,996 bp and a small single copy region of 12,876 bp. The genome contains a total of 131 genes, including 84 protein-coding genes, 8 ribosomal RNAs and 39 transfer RNAs. The GC content of the cp genome is 38.9%. Moreover, phylogenomic inference robustly placed *B. ventricosa* in the paleotropical woody bamboos lineage.

*Bambusa ventricosa* McClure, commonly called buddha bamboo, is a tropical woody bamboo with important ornamental value in the family Poaceae (Bambusoideae: Bambuseae) (Li et al. 2006). Unfortunately, the wild populations of *B. ventricosa* have been dramatically fragmented and decreased over the past decades, largely due to the agricultural activities. The genetic and genomic data of this bamboo are very scarce at present (Zhou et al. 2017). In this study, we characterized the complete chloroplast (cp) genome of *B. ventricosa* using the genome-skimming sequencing approach (Zhang and Chen 2016), which will provide essential resource for both conservation and utilization of this economically important bamboo.

Total genomic DNA was extracted from fresh leaves of *B. ventricosa* grown in Bamboo Garden of Sun Yat-sen University, in Guangzhou, Guangdong province of China. The voucher specimen was deposited at the Trees Herbarium of Sun Yat-sen University (accession number ZXZ181001). Illumina paired-end library was constructed and sequenced in Beijing Genomics Institute (BGI) in Shenzhen, China. The cp genome was assembled using CLC Genomics Workbench v7.5 (CLC Bio, Aarhus, Denmark), as conducted in previous studies (Wang et al. 2016). The assembled cp genome was annotated using DOGMA (Wyman et al. 2004), coupled with manual check and adjustment.

The cp genome of *B. ventricosa* (GenBank accession MH410121) is 139,460 base-pairs (bp) in size with high coverage (mean 316×), showing a typical quadripartite structure: one large single copy region of 82,996 bp and one small single copy region of 12,876 bp being separated by two inverted repeats of 21,794 bp. There are a total of 131 genes in the cp genome, including 84 protein-coding genes, 8 ribosomal RNA genes, and 39 transfer RNA genes. Nine protein-coding genes (*atpF*, *ndhA*, *ndhB*, *petB*, *petD*, *rpl16*, *rpl2*, *rps12*, *rps16*) contain one intron each while another one gene (*ycf3*) had two introns. Protein-coding regions (CDS) make up 43.2% of *B. ventricosa* cp genome and the overall GC content of this cp genome is 38.9%. As expected, the *B. ventricosa* cp genome displays a highly conserved genome structure of paleotropical woody bamboos (Bambuseae) (Wu et al. 2009).

Phylogenomic analysis was performed based on the complete cp genomes of 28 bamboos using RAxML v.8.2.8 (Stamatakis 2014). The resulted tree showed that *B. ventricosa* was clustered in the clade of paleotropical woody bamboos with high support value (Figure 1). The sister relationship of *B. ventricosa* and *B. oldhamii* was highly supported. The phylogenetic relationships among the sampled *Bambuseae* species were robustly resolved as well, in agreement with previous studies (Wang et al. 2018).

**Figure 1.**
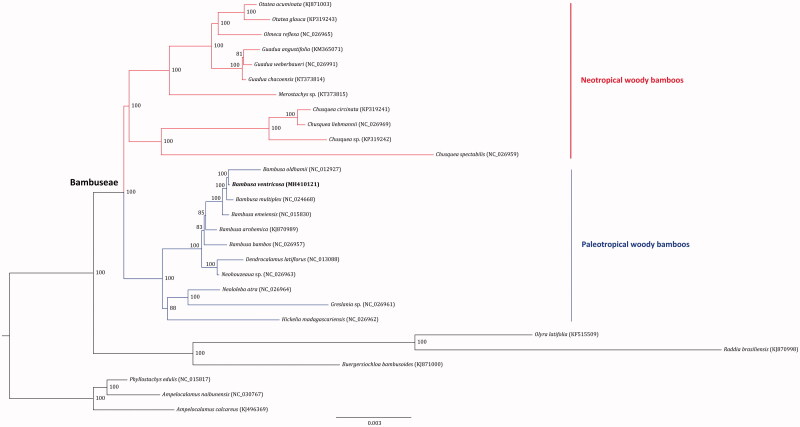
Maximum likelihood tree inferred from 28 bamboo chloroplast genomes. Colored branches indicate the paleotropical and neotropical Bambuseae lineages. The position of *Bambusa ventricosa* is shown in bold. Values associated with nodes are bootstrapping support values.
